# Advance Care Planning for Older People with Cancer and Its Implications in Asia: Highlighting the Mental Capacity and Relational Autonomy

**DOI:** 10.3390/geriatrics3030043

**Published:** 2018-07-20

**Authors:** Cheng-Pei Lin, Shao-Yi Cheng, Ping-Jen Chen

**Affiliations:** 1 Cicely Saunders Institute of Palliative Care, Policy & Rehabilitation, King’s College London, London SE5 9PJ, UK; cheng-pei.lin@kcl.ac.uk; 2 Department of Family Medicine, College of Medicine and Hospital, National Taiwan University, Taipei 100, Taiwan; scheng2140@gmail.com; 3 Department of Family Medicine, Kaohsiung Medical University Hospital, Kaohsiung Medical University, Kaohsiung 807, Taiwan; 4 Department of Division of Geriatrics and Gerontology, Kaohsiung Medical University Hospital, Kaohsiung Medical University, Kaohsiung 807, Taiwan; 5 Palliative Care Centre, Chi-Mei Medical Centre, Tainan 710, Taiwan

**Keywords:** ageing, cancer, advance care planning, mental capacity, relational autonomy

## Abstract

With dramatically increasing proportions of older people, global ageing has remarkably influenced healthcare services and policy making worldwide. Older people represent the majority of patients with cancer, leading to the increasing demand of healthcare due to more comorbidities and inherent frailty. The preference of older people with cancer are often ignored, and they are considered incapable of making choices for themselves, particularly medical decisions. This might impede the provision of their preferred care and lead to poor healthcare outcomes. Advance care planning (ACP) is considered an effective intervention to assist older people to think ahead and make a choice in accordance with their wishes when they possess capacity to do so. The implementation of ACP can potentially lead to positive impact for patients and families. However, the assessment of mental capacity among older adults with cancer might be a crucial concern when implementing ACP, as loss of mental capacity occurs frequently during disease deterioration and functional decline. This article aims to answer the following questions by exploring the existing evidence. How does ACP develop for older people with cancer? How can we measure mental capacity and what kind of principles for assessment we should apply? What are the facilitators and barriers when implementing an ACP in this population? Furthermore, a discussion about cultural adaptation and relevant legislation in Asia is elucidated for better understanding about its cultural appropriateness and the implications. Finally, recommendations in relation to early intervention with routine monitoring and examination of capacity assessment in clinical practice when delivering ACP, reconciling patient autonomy and family values by applying the concept of relational autonomy, and a corresponding legislation and public education should be in place in Asia. More research on ACP and capacity assessment in different cultural contexts and policy frameworks is highlighted as crucial factors for successful implementation of ACP.

## 1. Introduction

Ageing has become a key health policy issue worldwide due to the dramatic increase in the population of older people [1]. According to the World Report on Ageing and Health published by the World Health Organisation (WHO), the proportion of people aged 60 years or older in Europe, North America, Asia and Australia already reached 20–25% in 2015, while in sub-Saharan Africa, the population is still young. Japan is the only country where this proportion exceeded 30% in the same year. By 2050, more regions (e.g., China, North America, Europe and Russian Federation) will face a similar ageing situation to Japan in 2015 [1], and the number of older people is expected to be 1.5 billion, accounting for around 16% of the world’s population [2].

These older generations currently represent the majority of patients with cancer in the world, because ageing is one of the fundamental factors for the development of cancer [3]. The incidence of cancer has increased in older people due to their poor cellular repair mechanisms. According to the WHO, malignant cancer is the second leading cause of death in the world, and it was responsible for 8.8 million deaths in 2015. Globally, nearly one in six deaths are due to cancer [4]. This figure is growing gradually each year, leading to the increasing burden of healthcare systems for older people living with cancer from diagnosis to death.

The preferences of older patients with cancer are often ignored and they are considered lacking mental capacity for decision making simply because of their age and disease condition. Such patients might have no opportunity to speak for themselves in terms of desirable medical care at their end of life (EOL). Therefore, advance care planning (ACP) is considered an effective way to facilitate a person’s autonomy and enable provision of care and treatment aligned to an individual’s preferences. Evidence has shown that ACP can positively influence the elderly [5], nursing home residents [6] and people with dementia [7] and cancer [8]; whether a patient has been involved in ACP is considered a key indicator of high-quality hospice and palliative care [9]. However, research on ACP in older people with cancer is insufficient and lacking. This limitation is particularly important given the dramatic increase in the global number of older people with cancer and needs of palliative care for this population. This article aims to explore the following questions by applying the existing literature and evidence:

1. Current development and research work on ACP among older people with cancer in the world
2. The benefit of ACP for older people with cancer
3. The challenges and implications when promoting ACP in Asia
4. The recommendations for further clinical practice, research and policy convictions

## 2. Development of ACP

### 2.1. Definition and Meaning of ACP

ACP is a voluntary process that supports adults at any age or stage of health who possess mental capacity (the ability to make a decision for him- or herself) in understanding and sharing their personal values, life goals, and preferences regarding future (medical) care [10]. It is an ongoing process of assessment and communication among patients, family members, healthcare professionals and medical surrogates to reach a consensus on medical care for patients, and it consists of written documents such as advance directives/decisions (ADs) or advance statement (AS) [11,12,13]. ACP is usually used in the context of progressive illness and anticipated deterioration, and it greatly varies from general care planning [14].

### 2.2. Development and Legislation of ACP in the World

The rising popularity of ACP across the Western world reflects trends in palliative care towards emphasising the values of open awareness about EOL and dying issues and the promotion of patients’ autonomy [15]. The concept of a “living will” (a written document describing patient’s preferences about future care, which is also considered as a type of AD) was introduced in 1969 in the United States of America (USA) and then embedded in the USA legislation following two controversial cases about the withdrawal of life support from American people (Karen Quinlan and Nancy Cruzan) in the 1970s and 1980s [16]. To make the “living will” a legally binding document to ensure treatments meet patient’s preferences and minimise the uncertainty of healthcare decision making by families and medical team, the Patient Self-Determination Act [17] was adopted in the 1990s in the USA. This act mandates all patients be given information about their rights on decision making for future medical care by completing ADs or AS. Similar developments have been reported in other Western countries such as Australia, Canada, and United Kingdom (UK) as well as in Europe [16]. For example, in England, the End of Life Care Strategy was published in 2008 [18] and one of its aims is to ensure that healthcare services adequately meet EOL patients’ needs and preferences. Subsequently, relevant legislation or regulation was adopted to underpin the implementation of ACP in several countries (e.g., Mental Capacity Act 2005 [19] in England and Wales, Statute Law and Common Law in Australia [20], Mental Capacity Act 2008 in Singapore [21], and Patient Autonomy Act in Taiwan [22]). Recently, focus has moved from completing written documentations (e.g., ADs or AS) to an ongoing process of ACP discussion with patients for death preparation and future goals. Only completing an AD, which hypothesises specific clinical situations, will not necessarily improve a patient’s outcomes (e.g., quality of life and emotional distress) as it fails to capture the unique individualised care needs and recognise the availability of healthcare resources. An alternative approach, namely ACP, was developed in the 1990s to address this clinical dilemma for improving healthcare outcomes [23]. ACP is now widely used in Western countries, and this surge has influenced ACP development in other parts of the world. Examples of related activities include the promotion of ACP [24] and the legislation of the Patient Autonomy Act in Taiwan [22], the development of the ACP program “Let Me Talk” for nursing home residents in Hong Kong [25], a national survey of Japanese healthcare facilities’ perspectives on ACP [26], a study on family caregivers’ attitude and perspectives on ACP in Singapore [27] and EOL communication focusing on ACP with older adults in South Korea [28].

### 2.3. General Introduction of Current Research on ACP in the World

Previous studies have reported possible benefits of implementing ACP in patients with dementia, cognitive impairment [7] and cancer [8] as well as for older adults [29] including a reduction in aggressive medical care received, improvements in the quality of life, reduced hospital re-admission rates, increased use of palliative care, and a reduction in relatives’ levels of stress, anxiety and depression; these benefits make medical care cost-effective [5,7,8,11]. A systematic review (80% of evidence from the USA) conducted by Brinkman-Stoppelenburg et al. found that an ACP can positively impact the quality of EOL care, and outcomes of EOL care are improved when ACP is provided to patients in comparison with AD alone. This finding can also increase the compliance with a patient’s EOL care preferences [30].

The importance of ACP is currently supported by a host of Western countries such as Australia [31], United Kingdom [32] and USA [33] and endorsed by professional bodies, including the Australian [34], British [32] and American medical associations [35]. However, research on ACP in other cultures is relatively limited due to the delayed development of ACP compared with Western countries. For example, the concept of ACP is new to Asian society and underdeveloped. Introducing ACP to Asians may be a cultural challenge because discussions on EOL care and dying issues are sensitive and discouraged, so that older people are often not involved in an ACP discussion [24,25]. Results of studies on ACP in different cultures are inconsistent [36], which raised a crucial question: Can Western-oriented ACP intervention be translated to other cultures directly? A systematic review conducted by Zager and Yancy highlighted that different values, ethnicities and culture backgrounds affect attitudes to ACP and have powerful effects on EOL care decision making, suggesting that a culturally adapted ACP intervention is needed [36]. An ongoing systematic review supported this statement and found no empirical evidence on ACP among patients with advanced cancer out of Western countries to prove the effectiveness of ACP [37]. These results reveal the urgent need of conducting research on ACP among different ethnicities for testing cultural appropriateness and acceptability prior to the implementation.

## 3. Impact of ACP for Older People with Cancer

Studies have demonstrated the benefit of ACP for either older people or patients with cancer, but only a few specifically focused on older people with cancer [38]. According to the recent systematic review conducted by O’Caoimh and colleagues, ACP should be included in the survivorship care plan (SCP) for older people with cancer. But, no eligible paper has been found, thereby showing a lack of empirical evidence on the use of ACP within SCP for this population [38]. However, the effectiveness of ACP for older adults has already been determined by a systematic review conducted by Weather et al. This systematic review of randomised controlled trials (RCTs) was conducted to examine the effectiveness of ACP for older adults (>65 years) across health settings. Nine RCTs (seven studies conducted with community dwellings and two in a nursing home) were analysed, and improved process (e.g., ACP documentation), patient (e.g., knowledge of life-sustaining treatment and satisfaction with care) and family (e.g., less anxiety, stress, depression, and less bereavement) outcomes were found [5]. In addition, a systematic review of ACP for patients with advanced cancer conducted by Lin et al. reported similar findings, with the exception of an improvement in patient and family emotional distress [37].

Insufficient evidence on measuring quality of EOL care, death or dying experience and compliance with patient’s EOL wishes as primary outcomes were found. These are considered important indicators for high-quality EOL care [39] and strongly recommended to evaluate the effectiveness of ACP directly from users (patients) rather than proxy (care givers or family members). However, conducting a study involving older adult with cancer can be quite challenging due to their deteriorating disease condition and poor capacity for participation.

### 3.1. Facilitators and Barriers for ACP among Patients with Cancer or Older Adults

Some facilitators and barriers for initiating ACP discussion among patients with cancer or older adults were found in the works by Johnson et al. [8], Ke et al. [40] and Niranjan et al. [41]. In general, physician buy-in, patient readiness and prior experience of EOL care or decision making are considered facilitators. By contrast, restricted time, insufficient resources, social or personal taboos and institutional culture are deemed barriers for a successful ACP intervention. Most importantly, a family member’s view about ACP and stakeholders’ knowledge of ACP have crucial roles in ACP implementation.

Notably, ACP does not always result in positive effects because fear and distress of participants during the discussion regarding death and dying issues were found alongside with ACP implementation [8]. Nevertheless, the concept and definition of ACP are inconsistent in previous studies worldwide. Many studies treated ACP as AD documents rather than a mutual communication process between patients and healthcare professionals and focused only on the completion rate of a Do-Not-Resuscitate (DNR) forms or Physician Order for Life-Sustaining Treatment (POLST) forms (both are part of ADs) for refusing certain medical treatments. A systematic review conducted by Brinkman-Stoppelenburg et al. [30] reported that DNR order (39%) and ADs (34%) are the most often studied aspects of care. These do not necessarily improve a patient’s quality of EOL.

### 3.2. Mental Capacity as a Special Concern in ACP for Older People with Cancer

A lack of mental capacity for decision making and uncertainty of engaging ACP were also identified as barriers to undertake ACP for older people, particularly during their EOL [42]; these barriers compromised the quality of EOL care for patients with impaired mental capacity compared with those who only have cancer [43,44]. The process of informed consent needs to anticipate potential loss of capacity during the course of treatment and how a participant’s willingness to participate in certain care is upheld if they lose capacity. Therefore, assessment of mental capacity among people who might lose capacity in the future (e.g., older people and adults with cancer) is an inevitable process prior to the delivery of ACP intervention and should be embedded into clinical routine care to ensure the effectiveness for enhanced outcomes.

On the other hand, concerning the aspect of research, this particular population is often excluded from research given the difficulty researchers or clinicians have in judging whether they retain the ability to understand the content of research so that they can join voluntarily [42]. Including this group of people without consent prior to recruitment could be unethical as they are potentially vulnerable individuals with cognitive and functional impairment. Moreover, these individuals are urged to be included into studies on ACP as they are the potential stakeholders and representative subjects [42].

#### 3.2.1. Meaning of Mental Capacity and Principles to Protect a Person’s Right on Decision Making

The Mental Capacity Act (MCA) 2005, covering England and Wales, provided the statutory framework for people who have the capacity and want to make a decision in advance for themselves once they lose capacity in the future, and the principles of decision making for people who lack mental capacity. According to the MCA, a person who lacks mental capacity is defined as “a person who lacks capacity to make a particular decision or take a particular action for themselves at the time the decision or action needs to be taken” [19]. The decisions or actions can be day-to-day issues such as what to wear or what to eat during daily life or major medical decisions such as life-sustaining treatments or operations. This act is built on the following five principles to protect a person’s right on decision making [19]:

1. A person must be assumed to have mental capacity unless he or she is proven to lack capacity.
2. A person should not be considered as unable to make a decision unless all possible methods have been used without success.
3. A person should not be considered as unable to make a decision only because he or she made an unwise decision.
4. A decision made on a person’s behalf, who lacks capacity, should be in his or her best interest.
5. This act should be applied in a less restrictive manner of a person’s rights and freedom of action.

#### 3.2.2. ACP for People with Potentially Deteriorating or Fluctuating Mental Capacity

ACP is an opportunity to open early discussions about EOL care for older people or people with severe illness such as malignancies or cognitive disease such as dementia, whose mental capacity for decision making may degenerate gradually with the disease deterioration in the foreseeable future. People should be helped by any means possibly to allow them to make their own decisions before they are deemed as individuals who lack capacity. This principle has been strongly recommended in the latest report on ACP for people with dementia in all care settings proposed by the National Health Service (NHS) in England to guide the assessment of a person’s capacity on decision making [45]. The benefit of ACP for people with cognitive impairment and dementia has been confirmed by previous systematic reviews [7]; however, timing to assess the cognitive impairment of older adults and the criteria of excluding them from participating in an ACP still challenge healthcare professionals and impede clinical practices [43]. Therefore, some practical principles regarding mental capacity assessment and how to assist older adults with cancer make decisions for their future care are provided in the following section.

#### 3.2.3. How Should Capacity Be Assessed?

A two-stage assessment of mental capacity was suggested and promoted by MCA [19]. Firstly, any kind of impairment or disturbance affecting the way a person’s brain or mind works should be evaluated, regardless whether the impairment or disturbance is temporary or permanent. Secondly, whether the impairment cause the inability of a person’s decision making when he or she needs to make one should be assessed. Several questions could be asked for capacity assessment: Does the person understand the content of information about that he or she needs to decide? What are the reasons for his or her decisions, and what are the consequences? Is the person able to weigh the retained information? What is in the best interest for older people with cancer? Have all possible means been used to help him or her make a decision? Every person with disabilities (e.g., cognitive impairment) should be treated equally. A person’s lack of capacity should not be judged simply by their age, appearance, assumption about their condition and behaviour. This is strongly supported by MCA [19], NHS report on ACP for people with dementia [45] and Convention on the Right of Persons with Disabilities (CRPD) [46]. According to the previously published literature, there are validated assessment tools for testing an individual’s mental capacity, for example, MacArthur Competence Assessment Tool-Treatment (MacCAT-T) [47], Hopkins Competence Assessment Tool (HCAT) [48], Capacity Assessment Tool (CAT) [49], and Aid to Capacity Evaluation (ACE) [50]. The importance of these standardised tools was stressed by Sessums et al. and Heywood, and could be used as an evidence for capacity judgement in the court [51,52].

## 4. Challenges of Promoting and Implementing ACP in Asia: Taiwan and Singapore as Examples

### 4.1. Cultural Adaptation for the Concept of ACP and EOL Discussion

In Taiwan, although domestic studies regarding EOL care discussion and ACP with community older adults [24], patients with cancer [53] and healthcare professionals [54] have been conducted to investigate the congruence between patients’ EOL care preference and actual care received, the evidence and understanding in terms of how to initiate an effective EOL care or ACP discussion remain sparse and lacking. Most importantly, discussions with older adults about death, dying and related care issues are discouraged as a result of cultural taboo and an emotional distress of destroying patient’s hope [55,56]. Similar situations were also found in Singapore, as described by Ng et al. [27]. Thus, access to palliative care for older adults may be restricted due to the lack of opportunity to open a conversation in terms of EOL care among patients, families and healthcare professionals.

Evidence show that cultural backgrounds, religious beliefs, political convictions or past experience can shape a person’s values, beliefs and understanding towards healthcare, and this concept can affect the willingness of a person with full capacity in taking part in an ACP [19]. For example, Cheong et al. conducted a study on ACP in people with early cognitive impairment in Singapore and found that the majority of patients deferred decision making to their families; some considered ACP as irrelevant to their disease and unnecessary, while others presented avoidance and denial during ACP discussion [57]. Thus, ACP can be carried out in a very different manner under various cultural backgrounds, and cultural acceptance should also be considered during mental capacity assessment.

### 4.2. Involvement of Families and Physicians in ACP Is Crucial

In ACP, a culturally sensitive approach should be tailored for patients and their families in different cultural contexts. For example, people of Asian cultures often rely on family members to make decisions about their medical treatment for them, whilst personal will and preference is fundamental in Europe [36]. This collective decision-making style in Asian society is derived from the ingrained familism culture, which dominates Asian people’s daily lives [25,58]. The decision makers for critical medical treatments in Taiwan, for example, are usually not the patients themselves but the male (often the patient’s sons) or highly-educated family members. This phenomenon is caused by the patriarchal tradition and notion of filial piety [55]. A previous study in Taiwan found that DNR forms (a part of AD) for patients are mainly signed by family members (82.1%), and only a small proportion of patients (17.9%) signed the form themselves [59]. In Singapore, a qualitative study exploring the attitude and perceptions of Singaporeans regarding ACP in multi-cultural family-centric community from Menon et al. work reported similar findings that family members were deemed the decision-makers rather than the patients themselves [60]. This evidence revealed the deficiency of older people’s autonomy and the importance of involving family members in ACP discussions to reach a consensus when making decisions about a person’s EOL care in Asia. Furthermore, the delay of patient involvement in EOL care discussion before patients lose their capacity to make decisions might also explain this phenomenon.

The role of physicians in the ACP process is also vital in Asia. A qualitative study exploring knowledge, attitudes and perceptions of ACP in family caregivers of patients with advanced illness in Singapore reported that physicians, in general, are considered to possess requisite expertise to recommend appropriate medical care and are strongly trusted to advocate the best interests for patients [27]. The evidence stressed the cultural characteristics and importance of involving families and physicians in the ACP process. Truth-telling in terms of disease prognosis and EOL issues are also challenging for healthcare professionals in Asia. Taiwan and Singapore share the similarity that healthcare professionals typically disclose a patient’s poor prognosis to families rather than to the patients (especially older generation) to avoid depriving of hope from the latter and progress into overt depression [27,61]. However, patients might lose capacity to have a conversation in relation to preferred EOL care with families and medical teams if they age or their disease deteriorates.

### 4.3. Relational Autonomy as a Practical Approach in Asia

As to the challenges described in previous sections, we discuss whether the individualistic concept of autonomy in Western liberal viewpoint can be adapted into Asia? For example, many Asian people, especially those in Confucianism-influenced societies, consider the importance of family values and the “role specified relation oriented ethics” are prioritised compared to an individual’s opinions [36,62]. Meanwhile, insufficient health literacy among older adults with cancer is also found to influence the decision-making process [63]. In light of the above, older adults in Asia tended to authorise the right of decision-making to their families or significant others, leading to a tendency of endorsing principle of “beneficence” a favourable position that depreciate the respect for individual autonomy [62]. Therefore, a balanced multi-dimensional personhood, which places an individual in a socially embedded network with others, was proposed as a proper concept to explain the interdependence relationship between patients, family, medical professionals and community [64]. From our perspectives, the relational autonomy might be ideally used to assist Asian people, especially older adults, to share the decision-making on critical clinical situations with their families and important others due to the collectivism paradigm in Asia. According to Dove et al., relational autonomy has been considered as a solution to ethical and practical problems, and could bring positive impact on clinical practice and research [65]. However, it is noteworthy that the increase of complexity of decision-making process when adopting relational autonomy could be a drawback. Active endorsement and good interpretation for the wills and preferences of silent patients, and sufficient and effective communications with families and important others should be in place to minimise this threat. Besides, giving more space of participating decision process to family members or patient’s significant others in a patient’s medical decision-making is emphasised and recommended [65]. The ultimate goal of achieving patient autonomy in Asia might be not a clear black and white threshold regarding individualism or collectivism, but a shared decision-making with deliberated assistance from healthcare professionals to achieve the reconciling with medical and family paternalism [66].

### 4.4. Different Legislation Underpinning ACP in Asia

In Singapore, Common Law is adopted and the MCA was introduced in late 2009, assisting patients who might lose capacity due to different reasons in the future to make critical decisions regarding personal welfare and/or property and affairs. A failure of complying with MCA could be used as evidence in court. Physicians play a vital role in Singapore as they should enhance the ACP discussion, and also make decisions for patients based on their best interests if no Lasting Power of Attorney (LPA) was appointed to make a decision on behalf of the patients [21].

On the other hand, in Taiwan, the Patient’s Autonomy Act (PAA) was legislated in 2016 and will take effect in early 2019 [22] to underpin the delivery of ACP under the regulation of Civil Law, which is very different from Singapore. This legislation has made Taiwan the first country in Asia to develop a legitimate regulation for respecting a person’s medical autonomy. The aim of PAA is to re-emphasise an individual’s autonomy in medical decision-making in advance through a formal counselling process of ACP and provide the written documents (ADs) with a legally binding nature. Furthermore, the participation of close family member is compulsory in the ACP discussion, which boosts communication between patients and relatives, and the respect of family values, making relational autonomy possible in routine practice. A patient’s advanced decision in terms of life-sustaining treatments and EOL care can be archived on the government’s medical record system (National Insurance System), which is not interfered with by others. The uncertainty of the proper EOL care for patients could also be minimised. Once PAA officially takes effect in 2019, informing patients about their diagnosis, prognosis and treatment options directly will be a compulsory and statutory process, and this procedure might impact the way of breaking bad news in the healthcare system and the dynamics of medical decision-making model in Asian society.

Even though Taiwan and Singapore share some similar cultural backgrounds, the different legislation structures could form the ACP in very different ways. Considering local jurisdiction and regulation is requisite when developing a culturally appropriate ACP intervention for different cultures.

## 5. Recommendation

ACP, which concerns mental capacity assessment, should be embedded into clinical practice for older people with cancer to regularly examine their ability for decision making. Early intervention of ACP is crucial through the care process for this population, so that a sufficient time can be provided for older adults with cancer to enhance the patients’ understanding of the value and content of ACP. Subsequently, an informed decision should be made with assistance from family members and healthcare professionals when they still possess capacity. Patients’ mental capacity status should be regularly monitored and evaluated in clinical situations, and patients should be allowed and assisted to make a desirable choice about their care during the process of losing capacity.

Cultural difference is a crucial factor for successful ACP and should be considered carefully. A person’s value and belief about EOL care and dying issues might influence his or her acceptance of ACP discussion. The decision-making model in different cultures should be considered, and the background knowledge about local perspectives on EOL issues are also important prior to an ACP initiation. Furthermore, older people should be provided opportunities to engage in an ACP discussion, although some might lose the ability to do so. Subsequently, the completion of ADs should be encouraged among older people to ensure desirable care in the future. More studies about ACP for this vulnerable group in different cultures and area are necessary to enhance the evidence in clinical practice and research. Finally, corresponding legislation that underpins ACP intervention for older people with cancer is imperative, and a culturally adapted ACP discussion should be developed to accommodate people with different cultural backgrounds and ensure the effectiveness for positive outcomes. An Asian expert network on ACP consensus to construct a culturally sensitive framework for Asians has recently been launched and aims to achieve this goal.

## 6. Conclusions

ACP is a process of thinking ahead to treatment choices, goals of care and/or appointing another person to speak for oneself in the future. It has evolved from a legal, document-driven process to a process of engaging patients, families and medical professionals in conversations about wishes, goals and preferences with respect to care. An increasing number of patients and families have been reported to value ACP, but the majority of previous research has been undertaken in Europe or North America, with little evidence in the other side of the world. A lack of empirical evidence to prove the effectiveness of ACP among older people with cancer in the world is also noted. For this group of people, the assessment of mental capacity is a special concern and should be embedded into routine care prior to the ACP initiation. Cultural context on patient autonomy and local jurisdictions underpinning ACP should be considered when we engage older people with cancer into the ACP discussion. The concept and practice of relational autonomy might be ideal for the share decision-making in the ACP discussion between patients and their families or relatives due to the collectivism paradigm in Asia. More evidence regarding the cultural appropriateness and acceptance of ACP among older people with cancer in different cultures is urgently needed, as this population is expected to increase sharply in the near future. Moreover, a cultural-adapted consensus of ACP regarding the definition and recommendations for clinical practice should be a research priority in Asia.
